# Pressure-induced dramatic changes in organic–inorganic halide perovskites

**DOI:** 10.1039/c7sc01845b

**Published:** 2017-08-29

**Authors:** Xujie Lü, Wenge Yang, Quanxi Jia, Hongwu Xu

**Affiliations:** a Los Alamos National Laboratory , Los Alamos , NM 87545 , USA . Email: xujie@lanl.gov ; Email: hxu@lanl.gov; b Center for High Pressure Science and Technology Advanced Research , Shanghai 201203 , China; c Department of Materials Design and Innovation , University at Buffalo – The State University of New York , Buffalo , NY 14260 , USA . Email: qxjia@buffalo.edu

## Abstract

Organic–inorganic halide perovskites have emerged as a promising family of functional materials for advanced photovoltaic and optoelectronic applications with high performances and low costs. Various chemical methods and processing approaches have been employed to modify the compositions, structures, morphologies, and electronic properties of hybrid perovskites. However, challenges still remain in terms of their stability, the use of environmentally unfriendly chemicals, and the lack of an insightful understanding into structure–property relationships. Alternatively, pressure, a fundamental thermodynamic parameter that can significantly alter the atomic and electronic structures of functional materials, has been widely utilized to further our understanding of structure–property relationships, and also to enable emergent or enhanced properties of given materials. In this perspective, we describe the recent progress of high-pressure research on hybrid perovskites, particularly regarding pressure-induced novel phenomena and pressure-enhanced properties. We discuss the effect of pressure on structures and properties, their relationships and the underlying mechanisms. Finally, we give an outlook on future research avenues in which high pressure and related alternative methods such as chemical tailoring and interfacial engineering may lead to novel hybrid perovskites uniquely suited for high-performance energy applications.

## Abstract 2

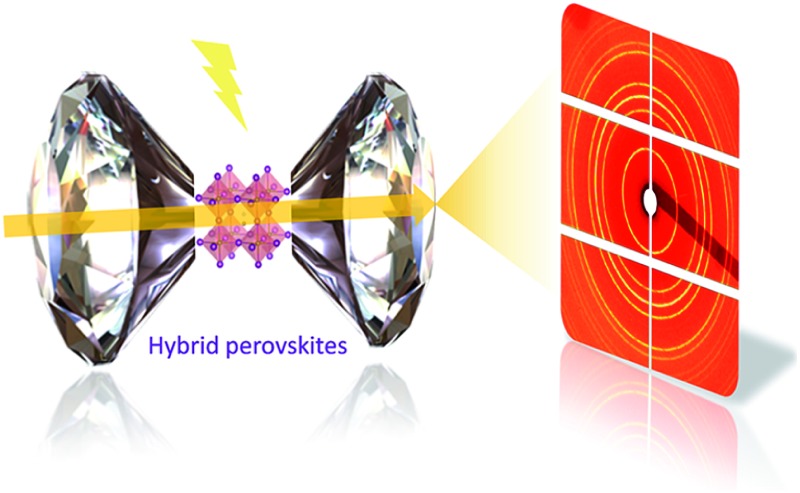
We summarise cutting-edge discoveries and provide insights into the important theme of halide perovskites using pressure as a tuning tool.

## 1. Introduction

During the past several years, the advent of perovskite solar cells (PSCs) based on organic–inorganic halide perovskites has revolutionized the prospect of next-generation photovoltaics, because PSCs show high energy conversion efficiencies and low processing costs.^1–5^ Since the first perovskite-based solar cell with a power conversion efficiency (PCE) of 3.8% was reported in 2009, unprecedentedly rapid progress has been made, achieving a certified PCE of over 22% recently.^6–13^ In addition, these hybrid perovskites have also been explored for use in optoelectronic applications such as photodetectors, light-emitting diodes, and lasers.^3,14–16^ The superior photovoltaic and optoelectronic performances have been attributed to their unique physical properties, including high optical absorption, small effective masses for electrons and holes, and long charge diffusion distances.^17–20^ Despite these advantageous attributes, challenges remain and they need to be addressed to advance their technological applications; these include low stability leading to device degradation and the use of lead, which incurs environmental concerns.^3,21–23^ These issues raise a crucial question about whether the impressively high device performances of hybrid perovskites can be realized for practical utilization, which requires both good stability and environmental friendliness. In order to answer this question there is a need for a better understanding of the structure–property relationships of hybrid perovskites.

Thus far, chemical manipulations have been employed to modify the structures, morphologies, and properties of hybrid perovskites.^24–27^ The major efforts have focused on optimizing their chemical compositions and increasing their crystallinity *via* crystal growth control, halide mixing, hetero-elemental combination, *etc.* In addition, different processing approaches such as one-step and sequential solution deposition,^7,28,29^ vapor-assisted solution processing,^30^ and solvent engineering^31^ have been developed to improve their properties and device performance. Although these chemical and processing methods have demonstrated great potential in optimizing this class of functional material towards higher performances, some challenging issues remain and novel strategies for material design/optimization are highly needed. Synthetic temperature has been proven to play a critical role in the modifications of the structures and properties of these hybrid perovskites.^32^ In parallel with temperature, pressure is another state parameter that provides an alternative dimension to effectively tune material properties by adjusting interatomic distances.

Pressure, as a fundamental thermodynamic variable, can dramatically alter the lattice and electronic configurations of materials, resulting in concomitant changes in their properties and functionalities. The developments in high-pressure science and technology in combination with in-laboratory and synchrotron-based probes have permitted a deeper understanding of a wide range of interesting phenomena, showing great potential for unearthing new material behaviors.^33^ In recent years, high pressure has been widely employed to modify the physical and chemical properties of functional materials and to further our understanding of the structure–property relationships.^34–38^ Moreover, high-pressure research enables the development of novel materials with emergent or enhanced properties, which otherwise cannot be achieved using traditional techniques.^39–42^ In the last few years, increasing numbers of studies have been reported to use high-pressure processing as an effective approach to adjust the structures and properties of organic–inorganic halide perovskites without changing their compositions and have revealed lots of intriguing pressure-induced phenomena.^37,43–59^ Until now, many previous reviews have dealt with halide perovskites, particularly regarding their functionalities and applications.^3–5,60–65^ In contrast, only a few review articles have discussed halide perovskites under pressure.^66–68^ These articles mainly discussed how pressure can alter their structural and physical properties, yet may lack systematic discussions into the common features and different aspects among these perovskites under high pressure. In this perspective, we provide a comparative analysis of pressure-induced dramatic changes in various characteristics of halide perovskites, and further summarize their common features, different behaviors and the underlying origins. We then seek to understand the effect of pressure on their structures and functionalities, as well as the structure–property relationships and associated mechanisms. We will, moreover, highlight the great importance of innovative strategies to potentially simulate external high pressures for further understanding and optimizing these hybrid perovskites, and also discuss the scientific issues, technological challenges, and potential directions from the perspective of materials design.

## 2. Basic knowledge of hybrid perovskites and high pressure research

The fundamental characteristics of organic–inorganic halide perovskites, including crystal structures and electronic, electrical and optical properties at ambient pressure, have been well summarized in previous reviews.^3–5,60–65^ Here, we will only highlight the information that is relevant to high-pressure research. In addition, we will provide a brief introduction to high pressure science in general, including pressure-induced dramatic changes in material properties, high-pressure characterization techniques, and the influence of experimental conditions.

### 2.1 Fundamental properties at ambient pressure

**Fig. 1:**
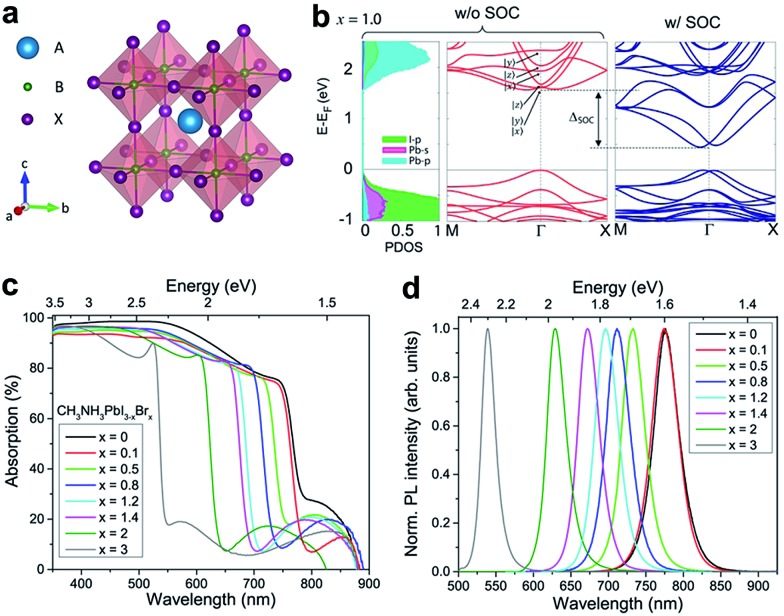
(a) Crystal structure of a perovskite ABX_3_, where A = MA^+^ or FA^+^; B = Pb^2+^, Sn^2+^, or Ge^2+^; and X = Cl^–^, Br^–^, I^–^ or mixtures thereof in organic–inorganic halide perovskites. (b) Electronic band structures of MAPbI_3_, the left panel corresponds to the projected density of states (PDOS) and the middle and right panels correspond to band structures without and with spin–orbit coupling (SOC), respectively. (c) UV-vis absorption spectra and (d) photoluminescence (PL) spectra of the mixed halide perovskites MAPbI_3–*x*_Br_*x*_. (b) is reproduced with permission from ref. 72, copyright 2015, American Chemical Society. (c and d) are reproduced with permission from ref. 73, copyright 2016, American Chemical Society.

Perovskite refers to a class of crystalline compound adopting the generic chemical formula ABX_3_, where each cation “B” has six nearest-neighbor anions “X” and cation “A” sits in a cavity formed by eight corner-shared BX_6_ octahedra (Fig. 1a). In the case of organic–inorganic hybrid perovskites, typically “A” is an organic cation [*e.g.* CH_3_NH_3_^+^ (MA^+^) or NH_2_CH=NH_2_^+^ (FA^+^)], “B” is a metal cation (Pb^2+^, Sn^2+^, or Ge^2+^), and “X” is a halogen anion (Cl^–^, Br^–^, or I^–^).^21^ The phase stability and crystal structures of perovskites can be deduced using the Goldschmidt’s tolerance factor *t* = (*R*_A_ + *R*_X_)/[√2(*R*_B_ + *R*_X_)] and an octahedral factor *μ* = *R*_B_/*R*_X_.^69^ The tolerance factor *t* is defined as the ratio of the A–X distance to the B–X distance in an idealized rigid-sphere model, where *R*_A_, *R*_B_ and *R*_X_ are the ionic radii of A, B and X, respectively. In the family of organic–inorganic halide perovskites, they can be stable to structural variants with tolerance factors of 0.81 < *t* < 1.11 and octahedral factors of 0.44 < *μ* < 0.90.^1,70^ If *t* lies in the range 0.9–1.0, a cubic structure is likely, while a deviated *t* value gives less symmetric tetragonal and orthorhombic structures.^1^ This feature will help us to understand pressure-induced structural evolution, where the A–X and B–X distances respond differently to pressure and thereby change the factors.

For current highly efficient PSCs, Pb is still the first choice for cation B, even though it is toxic. Replacing Pb with Sn can form similar perovskites with lower and more favorable bandgaps; these compounds, however, generally have lower stabilities due to the ease of oxidation from Sn^2+^ to Sn^4+^ (forming SnI_4_) in iodide perovskites.^71^ Starting from MAPbI_3_ as the archetypal system, it has been convincingly shown that all three of the lattice positions (A, B and X) can be fully substituted, giving rise to various series of mixed halide perovskites. The most studied mixed perovskite is MAPbI_3–*x*_X_*x*_, whose optical and electrical properties can be tuned by changing the relative proportions of the two halogens. Compounds of MAPbBr_3–*x*_Cl_*x*_ and FAPbX_3_, as well as their Sn and Ge perovskite analogues, have also been reported.^64^ In this perspective, we will review the high-pressure studies on MAPbBr_3_, MAPbI_3_ and their mixture, as well as MASnI_3_ and FAPbI_3_, since these have been the most investigated and some general conclusions can be derived.

Aside from some minor differences, the electronic structures of most organic–inorganic halide perovskites have similar characteristics. The valence band maximum (VBM) consists of a mix of an *n*p^6^ orbital from the halogen (*n* is the principal quantum number, *n* = 3, 4 and 5 for Cl, Br and I, respectively) and an *n*s^2^ orbital from the metal (*n* = 4, 5 and 6 for Ge, Sn and Pb, respectively); while the conduction band minimum (CBM) mainly consists of the empty *n*p^0^ orbital from the metal (Fig. 1b).^72,74^ The electronic states of organic cations in the A sites of the perovskite structure sit far away from the valence and conduction edges, thereby providing little direct contribution near the bandgap. However, the organic cations influence the lattice constants and thus indirectly affect the band structure. In addition, organic cations can affect the dielectric constants, hydrogen bonding (between the organic cation and the smaller halide anions), and inorganic octahedral distortion.^75,76^ Qualitatively, the band structure strongly depends on the symmetry of a given perovskite structure. A cubic structure is characterized by wider electronic bands, which indicate smaller effective masses and higher mobilities, and thereby render the cubic perovskites ideal candidates for technological applications.^37,64^ Halide perovskites are usually direct bandgap materials with high optical absorption coefficients.^1^ Their bandgap can be tuned over several hundred nanometers by changing the chemical compositions, such as the ratio of the constituent halides (Fig. 1c and d).^73^ This tunability offers a convenient approach to modify light absorption in solar cells, as well as open-circuit voltages. Nevertheless, it should be noted that the PL emission wavelength varies among samples prepared by different synthetic methods.^64^ A recent study proposed that the variation in PL wavelengths can be attributed to lattice strains in perovskite crystals.^77^ These local strains can be induced or simulated by applying external pressures, therefore, adjusting the pressure can be an effective method to tune the optical properties. Another advantageous attribute of hybrid perovskites is their high electron and hole mobilities, which can also be tuned by pressure.^37^

### 2.2 High pressure science and technology

Pressure provides a powerful tool for adjusting interatomic distances and bond lengths in materials, thereby effectively tuning the lattice and electronic structures as well as their properties and functionalities. High pressure treatment can significantly decrease the cell volume and increase the electronic density, which will result in novel physical properties and chemical reactivities.^78^ In recent years, high-pressure science and technology has developed from a small niche field and it is becoming a major dimension of materials science, and increasing numbers of discoveries and breakthroughs have been reported.^34–42^ For instance, the record high superconducting temperature of 203 K in high-pressure hydrogen sulfide has led to great excitement in the scientific community^79^ and nanotwinned diamond with unprecedented hardness and stability has been synthesized under high pressure.^41^ A key purpose of high-pressure research is to explore materials with useful properties, which can be preserved under ambient conditions for application. Even when the high-pressure phases cannot be preserved, the knowledge gathered at high pressures can often be used for ambient pressure synthesis with alternative methods.

The application of numerous dedicated synchrotron techniques to high-pressure research (particularly in combination with diamond anvil cells) has greatly enriched fundamental physics, chemistry, and materials science. High-pressure synchrotron techniques have rapidly developed. These include X-ray diffraction (XRD), which characterizes long-range crystal structures; the pair distribution function (PDF), which unveils short-range local bonding features at the atomic scale; X-ray emission spectroscopy, which provides information on filled electronic states; X-ray Raman spectroscopy, which monitors chemical bonding changes; nuclear resonant X-ray spectroscopy, which examines phonon densities of state; X-ray imaging, which provides information on hierarchical structures, dynamic processes and internal strains; *etc*.^80^ Integrating these state-of-the-art analytical methods with in-laboratory physical property measurements such as PL and absorption spectroscopy, electrical conductivity, and photocurrent measurement has enabled the *in situ* characterization of the structural, mechanical, electronic, optical, electrical, and optoelectronic properties under high pressure. The development of high-pressure science and technology in combination with synchrotron-based and in-laboratory methods has permitted a deeper understanding of a wide range of phenomena.^34–38^ On the other hand, one may notice that high-pressure results reported by different research groups are sometimes inconsistent. Such discrepancy is due, at least in part, to the different high-pressure experimental methods and conditions they used. A particular important aspect is the use of various pressure transmitting media, which determine the hydrostatic degree, strain level, pressure anisotropy and gradients. Non-hydrostatic conditions bring higher deviatoric stress and usually accelerate or even change pressure-induced reactions.^81–83^

## 3. Pressure-induced evolution of structures and properties

Recent investigations into pressure-induced behaviors of organic–inorganic halide perovskites have uncovered fascinating phenomena.^37,43–59^ Research on hybrid perovskites using high pressure as an external tuning means not only furthers our understanding of their structure–property relationships but also enables and guides the development of novel materials with emergent or enhanced properties that cannot be obtained by using traditional methods. For comparison, pressure-induced changes to the structures and properties of several halide perovskites are summarized in Table 1, from which we draw some general conclusions including common features and different behaviors. Common features include: (i) pressure-induced amorphization occurs during compression for all of the organic–inorganic halide perovskites studied, and the amorphous phases reverse back to crystalline perovskites after pressure release; (ii) pressure-induced PL variations are similar, where bond contraction broadens the band widths which leads to red shifts, while increased octahedral distortion and tilting at higher pressures cause blue shifts; and (iii) PL intensities generally weaken on compression and finally become undetectable when the pressure exceeds a certain threshold. Upon decompression, the PL peaks reappear. Different behaviors and their possible causes are: (i) intermediate high-pressure phases vary, which is possibly due to the different synthetic methods used to obtain the samples and the high-pressure experimental conditions used such as the pressure medium; and (ii) pressure-induced changes in conductivity exhibit opposite trends, which may not solely result from the different chemical compositions and initial structures of the hybrid perovskites and thus more investigations are needed to uncover the underlying mechanisms for electrical conductivity changes under high pressure. Detailed information is presented as follows.

**Table 1:** Comparison of pressure-induced changes to the structures and properties of several halide perovskites

	MAPbBr_3_		MAPbI_3_		MAPbI_1.2_Br_1.8_	MASnI_3_
	Ref. 43	Ref. 48	Ref. 44	Ref. 48	Ref. 48	Ref. 37
Crystal structure	Cubic *Pm*3*m*	Cubic *Pm*3*m*	Tetragonal *I*4/*mcm*	Orthorhombic *Fmmm*	Cubic *Pm*3*m*	Tetragonal *P*4*mm*
Cell parameters	*a* = 8.4413(6) Å	*a* = 5.9328(14) Å	*a* = 8.8648(6) Å, *c* = 12.6746(8) Å	*a* = 12.4984(7) Å, *b* = 12.5181(7) Å, *c* = 12.6012(8) Å	N/A	*a* = 6.240(1) Å, *c* = 6.227(2) Å
Experimental method and conditions	No pressure transmitting medium, up to 34 GPa at RT	Helium was used as the pressure medium, up to 48 GPa at RT	No pressure transmitting medium, up to 6.4 GPa at RT	Helium was used as the pressure medium, up to 46 GPa at RT	Helium was used as the pressure medium, up to 9.0 GPa at RT	No pressure transmitting medium, up to 30 GPa at RT
Pressure-induced structural evolution	*Pm*3*m* 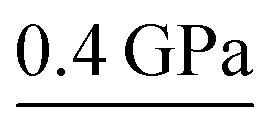 → *Im*3 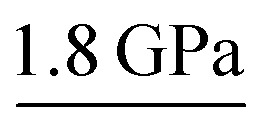 → *Pnma* 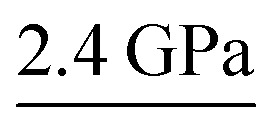 → amorphous	*Pm*3*m* 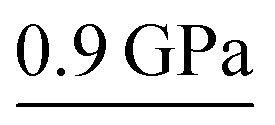 → *Im*3 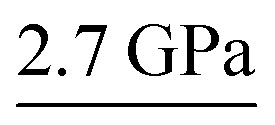 → amorphous	*I*4/*mcm* 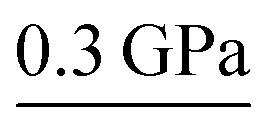 → *Im*3 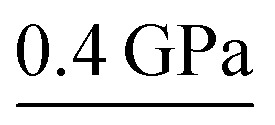 → *Immm* 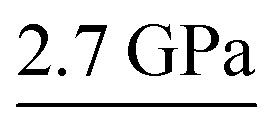 → amorphous	*Fmmm* 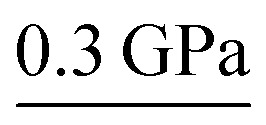 → *Im*3 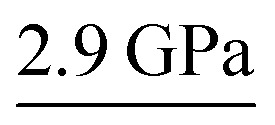 → amorphous	*Pm*3*m* 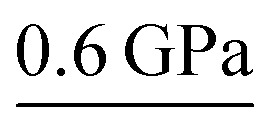 → *Im*3 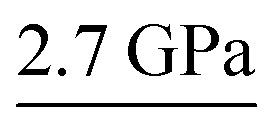 → amorphous	*P*4*mm* 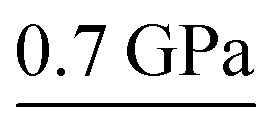 → *Pnma* 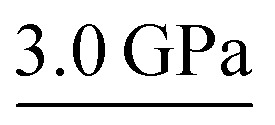 → amorphous
Optical properties	Red shift up to 1 GPa, followed by a blue shift	N/A	Red shift up to 0.4 GPa, followed by a blue shift	Red shift up to 0.3 GPa, followed by a blue shift	Red shift followed by a blue shift; a second PL peak appeared at 0.6 GPa	Photo-responsiveness enhanced after high pressure treatment
Electrical properties	Conductivity decreased by 10^5^ at 25 GPa	N/A	N/A	Conductivity increased by 10^3^ at 51 GPa	N/A	Conductivity decreased by 10^3^ at 12 GPa
Common features	(1) Pressure-induced amorphization during compression and the recovery of crystalline perovskites after the pressure is released					
	(2) Similar pressure-induced PL variations, where bond contraction broadens band widths leading to red shifts, while the increased octahedral distortion causes blue shifts					
	(3) PL intensities are weakened during compression and are finally undetectable; such a process is reversible upon decompression					
Different behaviors and possible causes	(1) Variation of intermediate high-pressure phases, which is possibly due to the synthetic methods used and associated experimental conditions such as the pressure medium					
	(2) Uncertainty of pressure-induced changes in conductivity. The contradictory results may not solely stem from the different chemical compositions and initial structures, and thus more studies are needed to uncover the underlying mechanisms of electrical conductivity changes under high pressure					

### 3.1 Structural phase transitions

MAPbX_3_ perovskites are the most popular subjects for high-pressure research because of their high performance for energy-related applications and better chemical stability compared to the Sn or Ge analogues. Structural phase transitions are the most studied aspect of hybrid perovskites under high pressure. Their lattice structures should be highly sensitive to external pressures, considering the flexible nature of the organic–inorganic hybrid frameworks. MAPbX_3_ forms different structures depending on the halogen anions. Under ambient conditions, MAPbBr_3_ crystallizes in a cubic structure of *Pm*3*m*,^43,48^ whereas MAPbI_3_ has been reported to crystallize in several tetragonal or orthorhombic structures with space groups of *I*4*cm*,^71^ *I*4/*mcm*,^44^ and *Fmmm*,^48^ where twinning incurs a complicated space-group assignment.^84,85^ As a result, in the subsequent high-pressure studies, different research groups used different space groups for MAPbI_3_ under ambient conditions.

**Fig. 2:**
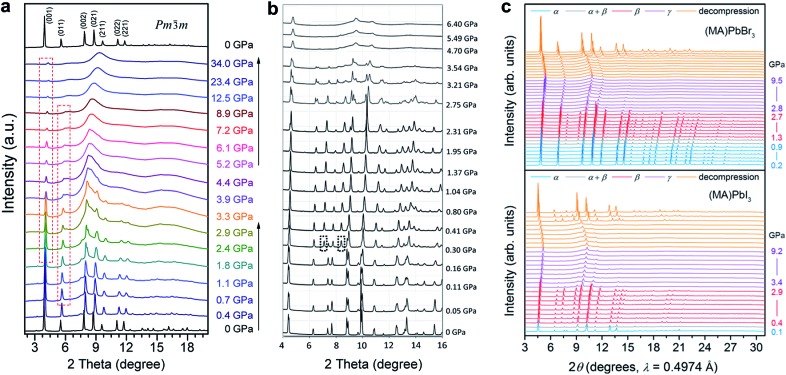
*In situ* synchrotron XRD patterns of (a) MAPbBr_3_ and (b) MAPbI_3_ collected at high pressures in DACs without a pressure transmitting medium. (c) XRD patterns of MAPbBr_3_ and MAPbI_3_ measured using helium as the pressure medium. Panel (c) shows more crystalline reflections even when the materials are partially amorphous; such a disparity can be explained by the different pressure media used. (a) is reproduced with permission from ref. 43, copyright 2015, American Chemical Society. (b) is reproduced with permission from ref. 44, copyright 2016, WILEY-VCH Verlag GmbH & Co. KGaA, Weinheim. (c) is reproduced with permission from ref. 48, copyright 2016, American Chemical Society.

Wang *et al.* reported the phase transitions of MAPbBr_3_ in a diamond anvil cell (DAC) under high pressures of up to 34 GPa, using *in situ* synchrotron powder XRD.^43^ As described above, MAPbBr_3_ crystallizes in a cubic perovskite structure (space group *Pm*3*m*) under ambient conditions. Two phase transitions were observed upon compression followed by an amorphization process (Fig. 2a). In other words, the cubic *Pm*3*m* structure transformed to another cubic structure of *Im*3 at ∼0.4 GPa by unit-cell doubling; then to an orthorhombic *Pnma* structure at around 1.8 GPa. The pressure-induced amorphization started at above 2 GPa and almost all of the diffraction peaks disappeared at 12.5 GPa. These phase transitions could be attributed to the tilting of PbBr_6_ octahedra and destroying of the long-range ordering of organic cations. Upon decompression, the amorphous phase reverted to the crystalline perovskite structure, exhibiting a memory effect.

Jiang *et al.* and Ou *et al.* reported the pressure-induced structural transitions of MAPbI_3_ using *in situ* synchrotron XRD, in combination with density functional theory (DFT) calculations.^44,46^ As shown in Fig. 2b, the initial tetragonal structure (*I*4/*mcm*) with lattice constants of *a* = 8.8648(6) Å and *c* = 12.6746(8) Å transformed to an *Im*3 space group with *a* = 12.4076(8) Å at ∼0.4 GPa, where octahedral tilting is at the same degree along all of the three axes. When the pressure reached 2.7 GPa, another phase transition to an orthorhombic structure was observed. Amorphization occurred with a further increase in pressure. Upon decompression, the sample remained amorphous until 0.58 GPa without the appearance of the orthorhombic polymorph. Instead, the *Im*3 phase appeared at 0.58 GPa, and the initial *I*4/*mcm* structure was recovered below 0.55 GPa. The authors stated that the recovered structure during decompression is dependent on the maximum applied pressure. The pressure-induced phase transition behavior of MAPbI_3_ is similar to that of MAPbBr_3_, in spite of their different compositions and initial structures. Szafrański and Katrusiak have performed a systematic study into the mechanism of pressure-induced phase transitions, amorphization, and absorption-edge shifting in MAPbI_3_.^47^ They demonstrated that the pressure-induced amorphization is triggered by an isostructural phase transition involving the frustrations in the inorganic framework, which is driven by strong interactions with disordered organic MA^+^ cations.

In order to examine the pressure-induced structural changes more precisely, Jaffe *et al.* conducted both powder and single-crystal XRD measurements of MAPbBr_3_, MAPbI_3_, and the mixed compounds MAPbI_3–*x*_Br_*x*_ (*x* = 0.6 and 1.8).^48^ For the sake of clarity, the authors defined the ambient pressure phase as α, the second phase under high pressure as β, and the third phase as γ. In the structure of the ambient pressure α phase of MAPbBr_3_ (space group *Pm*3*m*), all of the Pb–Br–Pb angles are 180°. Pressure tended to shorten the Pb–Br bonds, and the octahedral tilting occurred at the critical pressure for the α–β phase transition, forming a cubic *Im*3 phase at ∼0.9 GPa (Fig. 2c). Upon further compression above the α–β transition pressure, the Pb–Br–Pb angle decreased to 161.799(2)° at 1.0 GPa, which resulted from octahedral rotations. In the meantime, the Pb–Br distance was reduced from 2.9664(7) Å to 2.9406(1) Å over the same pressure range (ambient pressure to 1.0 GPa). Further volume reduction due to compression in the β phase happened with a combination of additional octahedral tilting and bond contraction. When the pressure was above ∼2.7 GPa, pressure-induced amorphization occurred, resulting in the formation of the amorphous γ phase. For MAPbI_3_, the structure under ambient conditions was refined to be orthorhombic with the space group *Fmmm* and *a* = 12.4984(7) Å, *b* = 12.5181(7) Å, and *c* = 12.6012(8) Å, which differs from the previously reported tetragonal symmetry. Two of the three iodide positions displayed non-ellipsoidal electron density distributions, indicating positional disorder. Although these two sites would have been equivalent in a tetragonal space group, the disorder was distinct in each site. With compression to 0.3 GPa, the phase underwent the α–β transition to form an *Im*3 phase; amorphization started at pressures above ∼2.9 GPa. With further compression, the amorphous γ phase remained stable up to the highest pressure applied. Both the MAPbBr_3_ and MAPbI_3_ samples reverted to their original phases, although some hysteresis occurred upon decompression. In addition, similar pressure-induced structural transitions were observed for the mixed-halide perovskites MAPbI_3–*x*_Br_*x*_ (*x* = 0.6 and 1.8). It should be noted that the phase transition to an orthorhombic structure prior to amorphization was not observed in this work. This may be because helium was employed as the pressure transmitting medium, which provided better hydrostatic conditions under high pressure (as discussed in Section 2.2). It is well known that non-hydrostatic conditions could facilitate pressure-induced structural transitions due to the effects of deviatoric stresses.^82,83^

Besides MAPbX_3_, the formamidinium (FA^+^, replacing MA^+^ at A sites in the perovskite lattices) and tin (Sn^2+^, replacing Pb^2+^ at B sites) analogues, such as FAPbI_3_, FAPbBr_3_, FASnI_3_, and MASnI_3_, have also been reported for potential photovoltaic and optoelectronic applications. The inorganic cation (B site) significantly affects the lattice and electronic structure; while the organic cation (A site) influences the lattice parameters, as well as the dielectric constants, hydrogen bonding (between the organic cation and halide anions), and octahedral distortion.^75,76^ The effects of the cations, both organic and inorganic, on the pressure-induced behaviors of halide perovskites are not clear yet, since only limited work has been reported so far. Generally, pressure-induced phase transitions followed by an amorphization are observed. In other words, the initial structures transform to new crystalline perovskites with varied intermediate phases, and finally become amorphous at higher pressures.^37,51,53,57^ The discrepancy is mostly due to their different compositions and initial structures, as well as the differences in high-pressure experimental conditions such as pressure transmitting media and pressure calibration.

Although pressure-induced structural transitions have been well investigated by *in situ* XRD measurements, the corresponding local bonding changes in the halide perovskite structures are not fully characterized. This is partially due to the complexity of the organic–inorganic hybrid structures, their sensitivity to focused laser illumination (which would cause irreversible degradation), and the resolution limits of analytical methods (*e.g.* Raman spectroscopy) used under high pressure. Hence, more suitable experimental methodologies, such as real space PDF analysis in combination with theoretical calculations, are needed for further studies.

### 3.2 Electronic and physical property evolution

Having examined the structural evolution of organic–inorganic halide perovskites as a function of pressure, we turn to describe the corresponding pressure-induced evolution of electronic and physical properties.

**Fig. 3:**
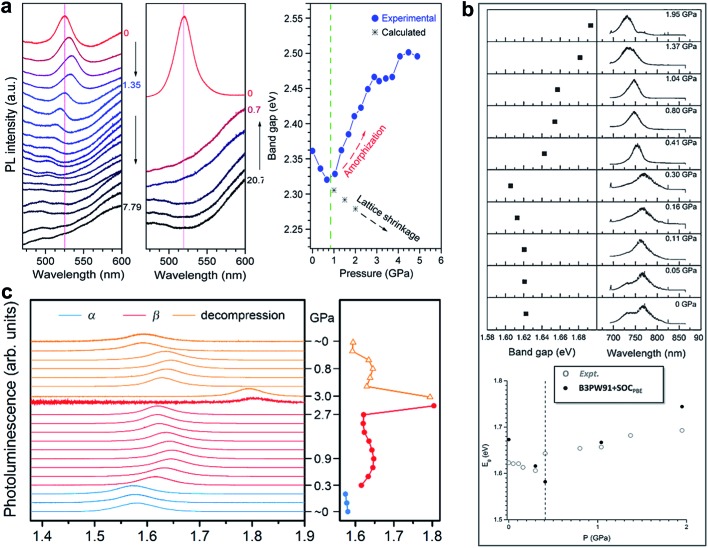
PL spectra and the derived bandgaps under various pressures of (a) polycrystalline MAPbBr_3_, (b) polycrystalline MAPbI_3_, and (c) single-crystalline MAPbI_3_. (a) is reproduced with permission from ref. 43, copyright 2015, American Chemical Society. (b) is reproduced with permission from ref. 44, copyright 2016, WILEY-VCH Verlag GmbH & Co. KGaA, Weinheim. (c) is reproduced with permission from ref. 48, copyright 2016, American Chemical Society.

Matsuishi *et al.* and Wang *et al.* studied pressure-induced variations in the bandgap of polycrystalline MAPbBr_3_ by an *in situ* analytical technique with DACs, in combination with first-principles calculations.^43,86^ The bandgaps were derived from both PL spectra and band structure calculations, as shown in Fig. 3a. On compression, the bandgap showed a red shift below 1 GPa followed by a blue shift at higher pressures. With a further increase in pressure, the PL peaks became weaker and finally undetectable due to the enhanced non-radiative processes in the amorphous structure. Upon decompression to ambient pressure, the sample regained the emission signals, accompanied by recrystallization to the original perovskite structure. On the other hand, Jiang *et al.* examined the pressure-induced bandgap changes of polycrystalline MAPbI_3_ using both PL spectroscopy and DFT calculations (Fig. 3b).^44^ Similar behavior was revealed, that is, the PL emission showed a gradual red shift during compression up to 0.3 GPa, followed by a blue shift at 0.4 GPa, which corresponds to the pressure-induced phase transition. Eventually, the PL spectra vanished at above 2.7 GPa. Consistent results of PL spectral evolution have also been reported in single-crystalline MAPbI_3_ and the mixed perovskites (*e.g.* MAPbBr_1.8_I_1.2_),^48^ as shown in Fig. 3c. The pressure-dependent PL variations in these halide perovskites are similar, in spite of their different initial bandgaps and crystalline structures. These changes in PL emission energy with pressure correlate well with structural evolution, that is, bond contraction results in larger orbital overlap and this consequently increases the band dispersion and reduces the bandgap, while the pressure-induced octahedral distortion and tilting decrease the orbital overlap and increase the bandgap. These results, together with the pressure-induced structural changes, are summarized in Table 1 for better comparison.

**Fig. 4:**
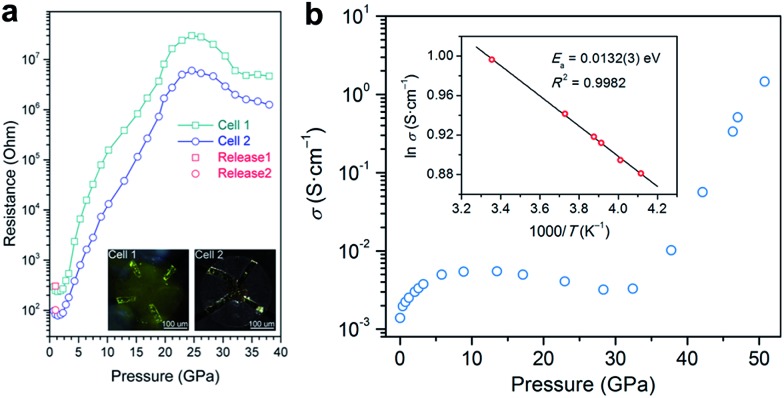
(a) Electrical resistance of MAPbBr_3_ at various pressures. The inset in (a) displays microphotographs of samples in two DACs with Au electrodes. (b) Electrical conductivity of MAPbI_3_ as a function of pressure. The inset in (b) shows the Arrhenius fit of the temperature dependence of conductivity at 51 GPa, which gives a low activation energy of 13.2 meV. (a) is reproduced with permission from ref. 43, copyright 2015, American Chemical Society. (b) is reproduced with permission from ref. 48, copyright 2016, American Chemical Society.

Electrical conductivity is one of the most important parameters for photovoltaic materials. Fig. 4a shows the electrical resistance change of MAPbBr_3_ as a function of pressure, which was recorded by Wang *et al.* ^43^ The resistance decreased first, which is usually caused by the increase in orbital overlap (*i.e.* increased band dispersion) and density of the sample upon compression, then the resistance increased sharply by five orders of magnitude from 2 to 25 GPa, which the authors attributed to pressure-induced disordering (amorphization). On the other hand, a very different behavior for the pressure-induced conductivity change in MAPbI_3_ was reported by Jaffe *et al.* ^48^ As shown in Fig. 4b, conductivity increased and reached a plateau on compression to about 5 GPa, and then decreased slightly upon further compression to 30 GPa. At higher pressures of up to 51 GPa, a dramatic increase in conductivity by two orders of magnitude was observed. The authors attributed the sharp increase to the reduced carrier effective mass and provided further evidence for the pressure-induced metallization of MAPbI_3_ in their following work.^55^ More recently, a dramatic increase in the electrical conductivity of FAPbI_3_ was reported during compression and a semiconductor-to-metal transition was observed at 53 GPa and 41 GPa for α- and δ-FAPbI_3_, respectively.^53^ Such a big discrepancy between the aforementioned studies may not just result from their different chemical compositions and initial structures, and thus further investigations are required to uncover the underlying mechanism of electrical conductivity changes under high pressure.

### 3.3 Enhanced properties induced by high-pressure treatment

In addition to improving the fundamental understanding of structure–property relationships, another primary motivation of high-pressure research is to see if these treatments can enhance and optimize the properties and functionalities of hybrid perovskites for their practical applications. The knowledge gained would lead to alternative routes for the design and synthesis of high-pressure phases with superior properties under ambient conditions. For instance, pressure-induced higher superconducting transition temperatures (*T*_c_) in iron-based superconductors have been obtained by chemical substitutions to simulate pressure effects.^87,88^ Investigation into functional materials using high pressure as a tuning tool has stood out as one of the key research directions that an increasing number of scientists are pursuing.

**Fig. 5:**
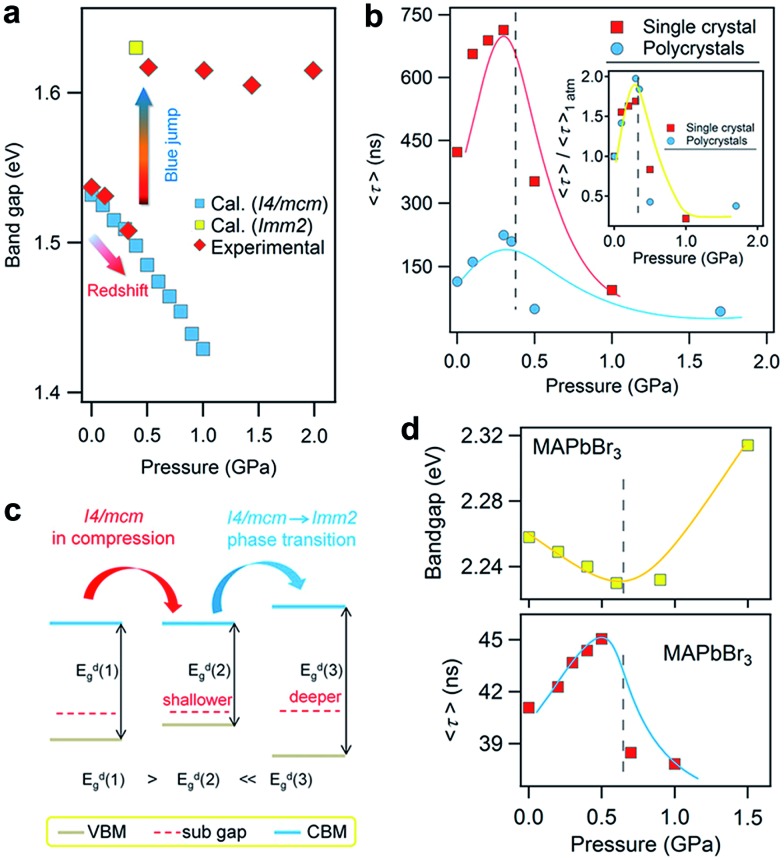
(a) The pressure-driven bandgap evolution of MAPbI_3_ with both experimentally measured data and theoretically calculated values. (b) The pressure dependence of the mean carrier lifetime *τ* for both single-crystalline and polycrystalline MAPbI_3_ with peak *τ* values at 0.3 GPa. The inset displays the normalized results. (c) Schematic illustrations of the band edge shifts and carrier-lifetime prolongation under mild pressure. As the bandgap narrows, the subgap approaches the VBM and makes the trap state shallower, contributing to the larger carrier lifetime. (d) The pressure-induced changes in the bandgap and carrier lifetime of MAPbBr_3_. Reproduced with permission from ref. 49.

Recently, Kong *et al.* investigated the pressure-induced bandgap evolution of MAPbI_3_ and MAPbBr_3_ together with changes in carrier lifetime, revealing a synergistic enhancement in both bandgap narrowing and carrier-lifetime prolongation under mild pressures of ∼0.3 GPa.^49^ At ambient pressure, the bandgap of MAPbI_3_ was determined to be 1.537 eV.^21^ During compression, a red shift of the bandgap to 1.507 eV was first observed at 0.32 GPa (Fig. 5a). As mentioned previously, the VBM of MAPbI_3_ consists of the I 5p^6^ and Pb 6s^2^ orbitals, while the CBM consists of the Pb 6p^0^ orbital. Note that as the pressure slightly increased, the shortening of bond lengths dominated the lattice change. As such, the coupling of the I p and Pb s orbitals increased and pushed the VBM up in energy. On the other hand, the CBM is mostly a non-bonding localized state of Pb p orbitals, which is not sensitive to bond length changes. Therefore, the pressure-induced narrowing of the bandgap mainly results from the increase in the energy of the VBM. It is worth noting that if the perovskite could be retained in its original phase, the pressure-induced bandgap narrowing would continue with the shortening of bond lengths, as revealed by first-principles calculations (Fig. 5a). Experimentally, a blue jump in the bandgap was detected along with the pressure-induced phase transition, where the Pb–I–Pb angle decreased and the orbital overlap reduced.

Simultaneously, the authors conducted *in situ* time-resolved PL measurements under high pressure on these perovskites to study the effect of pressure on their carrier lifetime *τ* (Fig. 5b). At ambient pressure, a single-crystal of MAPbI_3_ shows a carrier lifetime *τ* of 425 ns. With increasing pressure, the carrier lifetime increased to 658 ns at 0.1 GPa and then reached a peak value of *τ* = 715 ns at 0.3 GPa. Note that this pressure is consistent with that at which the narrowest bandgap is obtained. Higher pressures induce a phase transition and sharply reduce the carrier lifetime. Polycrystalline MAPbI_3_ exhibits similar behavior under high pressure, except that the *τ* values are smaller than those of its single-crystal counterpart. Fig. 5c schematically elucidates the pressure-induced change in the bandgap and the increase in carrier lifetime. As shown in the figure, the trap states, which sit in the subgap close to the VBM, become even shallower under a mild pressure. In addition, the same experiments were performed on its bromide analogue MAPbBr_3_ and similar behavior was observed (Fig. 5d), suggesting that the behavior of simultaneous bandgap narrowing and carrier-lifetime prolongation might be a general behavior in organolead halide perovskites. Hence, it is conceivable that these property enhancements achieved under such a mild pressure of ∼0.3 GPa could also be realized under ambient conditions through mechanical or chemical means; for example, chemical pressure exerted through ion substitution may modulate the related structure in a manner similar to external pressure. In contrast, Wang *et al.* reported an opposite trend in the change of the lifetime of MAPbI_3_ thin films in that the carrier lifetime drastically decreases with increasing pressure.^89^ Such a discrepancy likely arises from the very different samples used. Kong *et al.* studied single- and poly-crystals, while Wang *et al.* investigated thin films on fused silica glass where the substrate would have a significant influence. Moreover, their high-pressure experimental setups were different, such as the pressure media, which could also lead to different results. In addition, it is worth noting that the band structure of MAPbI_3_ under ambient conditions, direct or indirect, is still a matter of debate.^89–91^

It has been well demonstrated that pressure can effectively tune or even improve the properties of organic–inorganic halide perovskites by altering their crystal structures. However, one particular un-answered question is whether the pressure-induced unique properties can be retained in the samples after the pressure is released. In other words, what are the differences between the original and pressure-induced phases in terms of the structure and properties? From this point of view, Lü *et al.* compared the structures and optoelectronic properties of a lead-free halide perovskite MASnI_3_ before and after high-pressure treatment.^37^

**Fig. 6:**
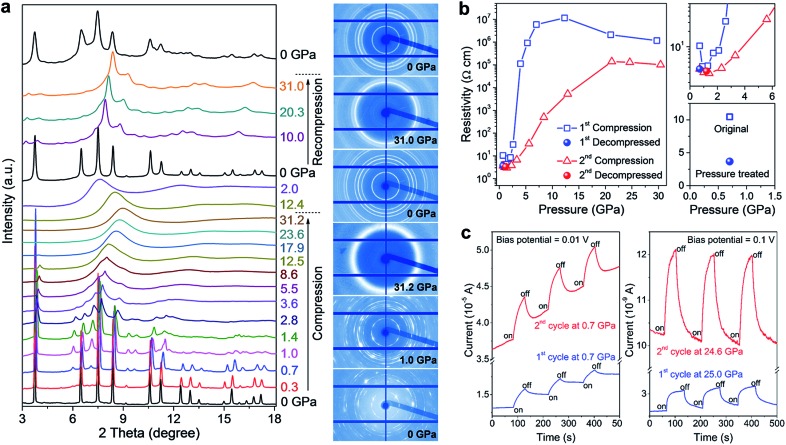
(a) *In situ* structural characterization of MASnI_3_ under high pressure. The left panel shows the XRD patterns collected during two sequential compression–decompression cycles and the right panel shows the raw XRD images at six selected pressures. (b) Pressure-induced resistivity evolution in the two compression–decompression cycles and a comparison of the resistivities before (open square) and after (solid sphere) high-pressure treatment. (c) Photocurrents of MASnI_3_ before (first cycle) and after (second cycle) pressure treatment, at a low pressure of 0.7 GPa (left panel) and at a high pressure of 25 GPa (right panel). The blue line shows the first cycle and the red line shows the second cycle. Reproduced with permission from ref. 37, copyright 2016, WILEY-VCH Verlag GmbH & Co. KGaA, Weinheim.

Impressively, the authors uncovered significant improvements in the structural stability, electrical conductivity, and photo-responsiveness of the perovskite *via* pressure-induced amorphization and recrystallization processes.^37^ To observe these phenomena, they carried out *in situ* XRD, Raman spectroscopy, electrical resistivity, and photocurrent measurements in a DAC during two sequential compression–decompression cycles. In the first cycle, the hybrid perovskite underwent pressure-induced amorphization at ∼3 GPa, followed by recrystallization to the perovskite structure upon pressure release. During the second compression process, surprisingly, no amorphization was observed even at above 30 GPa (Fig. 6a). It can thus be concluded that the pressure-treated perovskite possesses enhanced stability even though it retains a similar crystal structure to the original phase. *In situ* resistance measurements revealed a three-fold increase in the electrical conductivity of the pressure-treated MASnI_3_ in comparison to that of the pristine sample (Fig. 6b), which is partially due to its higher electron mobility as confirmed by first-principles calculations. Photocurrent measurements also demonstrated a substantial enhancement in the visible-light responsiveness of the perovskite after high pressure treatment (Fig. 6c). The mechanisms underlying the enhanced structural stability and the associated property improvements were systematically discussed using both theoretical arguments and experimental evidence; these include higher crystallographic symmetry, more uniform grain sizes and microstructural modifications induced by pressure treatment. These findings may provide a new perspective on the understanding of the fundamental relationship between the local structures and optoelectronic properties of halide perovskites, and also open up an alternative method to optimize these materials for the development of high performance photovoltaic and optoelectronic devices.

**Fig. 7:**
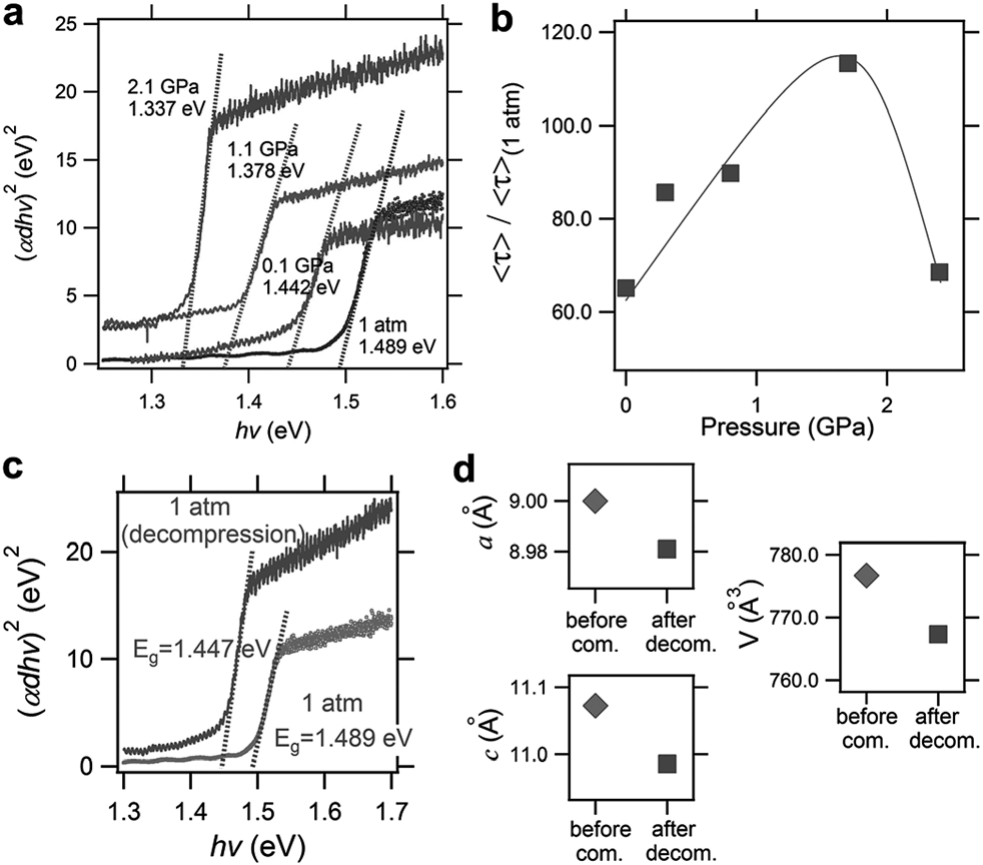
Pressure-induced enhancement of FAPbI_3_. (a) Optical absorption spectra during compression from ambient pressure to 2.1 GPa, showing a redshift of the bandgap from 1.489 to 1.337 eV. (b) The pressure dependence of the carrier lifetime. The maximum value was observed at 1.7 GPa where an increase of 120% was observed. (c) A comparison of the absorption spectra before and after pressure treatment, showing the partial retainability of bandgap narrowing. (d) A comparison of the lattice parameters and cell volume before and after pressure treatment. Reproduced with permission from ref. 56, copyright 2017, WILEY-VCH Verlag GmbH & Co. KGaA, Weinheim.

More recently, Liu *et al.* reported the improved properties of FAPbI_3_ after pressure treatment, where a pressure-induced decrease of the bandgap from 1.489 to 1.337 eV (Fig. 7a) and an increase in the carrier lifetime of 120% (Fig. 7b) were demonstrated.^56^ Importantly, these improvements are partially retained after the complete release of pressure (Fig. 7c). The authors attributed the retainability of the bandgap narrowing to the non-reversibility of lattice shrinkage in response to the applied pressure (Fig. 7d). Although the tiny samples treated under high pressure in DACs are not suitable for practical applications, these findings do provide valuable knowledge for the design and synthesis of high performance materials. For example, large-volume press facilities can be used to prepare larger-size samples. Furthermore, one may modify the performance of functional materials by exploring alternative approaches to high pressure, *e.g.*, *via* interfacial engineering, to generate local strains in hybrid perovskite films to achieve desired properties under ambient conditions.

## 4. Conclusions and outlook

Although high-pressure research on organic–inorganic halide perovskites is still in its infancy, unprecedented progress has been achieved. Pressure has been successfully used to modify the structures and properties of these hybrid perovskites, where various *in situ* analytical methods have been employed under high pressure. For instance, synchrotron XRD, Raman, PL, and UV-vis-NIR absorption spectroscopy, electrical resistance, and photocurrent measurements have been utilized to examine the pressure-induced evolution of the structural, mechanical, optical, electrical, and optoelectronic properties. One expects that more high-pressure characterization methods will be introduced. For example, PDF analyses of synchrotron X-ray and neutron total scattering data can be used to investigate the characteristics of pressure-induced amorphous structures of hybrid perovskites and pump-probe ultrafast spectroscopy may be used to study the photo-electronic processes and understand the charge carrier dynamics of these materials under high pressure. The burgeoning field of high-pressure research on hybrid perovskites can potentially be expanded by examining more comprehensive systems. Promising candidates include lead-free metastable halide perovskites, such as Sn, Ge, Sb and Bi analogues, *e.g.* FASnX_3_, MAGeX_3_, MABiSX_2_, as well as two-dimensional halide perovskites, *e.g.* (C_4_H_9_NH_3_)_2_(CH_3_NH_3_)_2_Pb_3_I_10_ and (C_4_H_9_NH_3_)_2_(CH_3_NH_3_)_3_Pb_4_I_13_.

As discussed above, high-pressure research can greatly facilitate our understanding of the basic science and the structure–property relationships of hybrid perovskites. The ultimate goal of applying high pressure to these materials is to further optimize their properties and functionalities for tailored applications in photovoltaic and optoelectronic devices. Therefore, more investigations, such as those on the difference in the thermodynamic stability of perovskites before and after high-pressure treatment, are needed to achieve a more comprehensive and in-depth understanding. Then, the knowledge gained in this high-pressure research can be used to guide the design and synthesis of unique high-pressure phases *via* alternative routes under ambient conditions. For example, (i) the external pressures can be mimicked by intentional chemical tailoring, where the introduction of ions with different sizes can effectively modify the internal pressure and stabilize high-pressure polymorphs. The effects of chemical pressure have already been demonstrated to simulate those of external pressure in iron-based superconductors to realize higher *T*_c_. (ii) Interfacial engineering can also be used to generate/tune local strains in films in order to simulate the effects of external pressure on hybrid perovskites and therefore obtain desired properties. These combined chemical-pressure and interface-pressure strategies may produce novel methods for the development of new and better hybrid perovskites for advanced energy applications.

Despite its tremendous progress, high-pressure research on hybrid perovskites still faces a number of challenges. (i) High-pressure probing usually involves a small piece/area of sample (micron scale); non-uniformity of the sample brings more uncertainties and increases its complexity. For example, some properties (*e.g.* electrical conductivity) are highly related to the exposed facets of perovskite crystals. (ii) Hybrid perovskites are sensitive to moist air and focused laser illumination, which causes irreversible degradation and renders difficulties in obtaining accurate results. (iii) The resolutions of analytical methods are sometimes not high enough under high pressure. Hence, more suitable experimental methodologies combined with theoretical calculations are highly desired. Through an iterative process, the experiments will yield reliable input parameters for simulation and modeling, while the calculations will provide mechanistic explanation and a deeper understanding of the experimental results.

In short, organic–inorganic halide perovskites not only offer exciting potential for next-generation photovoltaics and optoelectronics, but also provide intriguing systems that will foster new interdisciplinary research that integrates physics, chemistry, materials science, and high-pressure research.

## Conflicts of interest

There are no conflicts to declare.
